# NEDDylation Is Essential for Kaposi’s Sarcoma-Associated Herpesvirus Latency and Lytic Reactivation and Represents a Novel Anti-KSHV Target

**DOI:** 10.1371/journal.ppat.1004771

**Published:** 2015-03-20

**Authors:** David J. Hughes, Jennifer J. Wood, Brian R. Jackson, Belinda Baquero-Pérez, Adrian Whitehouse

**Affiliations:** 1 School of Molecular and Cellular Biology, Faculty of Biological Sciences, University of Leeds, Leeds, United Kingdom; 2 Astbury Centre for Structural Molecular Biology, Faculty of Biological Sciences, University of Leeds, Leeds, United Kingdom; Oregon Health and Science University, UNITED STATES

## Abstract

Kaposi’s sarcoma-associated herpesvirus (KSHV) is the causative agent of Kaposi's sarcoma (KS) and primary effusion lymphoma (PEL), which are aggressive malignancies associated with immunocompromised patients. For many non-viral malignancies, therapeutically targeting the ubiquitin proteasome system (UPS) has been successful. Likewise, laboratory studies have demonstrated that inhibition of the UPS might provide a promising avenue for the treatment of KSHV-associated diseases. The largest class of E3 ubiquitin ligases are the cullin-RING ligases (CRLs) that are activated by an additional ubiquitin-like protein, NEDD8. We show that pharmacological inhibition of NEDDylation (using the small molecule inhibitor MLN4924) is cytotoxic to PEL cells by inhibiting NF-κB. We also show that CRL4B is a novel regulator of latency as its inhibition reactivated lytic gene expression. Furthermore, we uncovered a requirement for NEDDylation during the reactivation of the KSHV lytic cycle. Intriguingly, inhibition prevented viral DNA replication but not lytic cycle-associated gene expression, highlighting a novel mechanism that uncouples these two features of KSHV biology. Mechanistically, we show that MLN4924 treatment precluded the recruitment of the viral pre-replication complex to the origin of lytic DNA replication (OriLyt). These new findings have revealed novel mechanisms that regulate KSHV latency and reactivation. Moreover, they demonstrate that inhibition of NEDDylation represents a novel approach for the treatment of KSHV-associated malignancies.

## Author Summary

Kaposi’s sarcoma-associated herpesvirus (KSHV) causes Kaposi’s sarcoma (KS) and primary effusion lymphoma (PEL), often fatal malignancies afflicting HIV-infected patients. Previous research has shown that blockade of the ubiquitin proteasome system (UPS, a normal quality control pathway that degrades cellular proteins) is able to kill KSHV-infected lymphoma cells. A large component of the UPS is made up by the protein family known as the cullin-RING ubiquitin ligases (CRLs), which are activated by NEDD8 (a process known as NEDDylation). Recently, an inhibitor of NEDDylation (MLN4924) was developed and is currently in clinical trials as an anti-cancer drug. As NEDDylation has not been investigated for many viruses, we used this to compound examine its importance in KSHV biology. Firstly we show that NEDDylation is essential for the viability of KSHV-infected lymphoma cells, and MLN4924 treatment killed these cells by blocking NF-κB activity (required for KSHV latency gene expression and KSHV-associated cancer). Furthermore, we show that NEDDylation is required for KSHV to replicate its genome, a critical step in the production of new virus particles. Therefore, this research has identified a novel molecular mechanism that governs KSHV replication. Furthermore, it demonstrates that NEDDylation is a viable target for the treatment of KSHV-associated malignancies.

## Introduction

The ubiquitin-proteasome system (UPS) and associated pathways are rapidly becoming accepted as major therapeutic targets for the treatment of malignancy [1], which potentially include those associated with oncogenic viruses. Additionally, small molecule inhibitors have been successfully used for dissecting the biological roles of these intriguing pathways, which is critical for our understanding of their mechanisms of cytotoxicity. Indeed, inhibition of the UPS is cytotoxic to Kaposi’s sarcoma-associated herpesvirus (KSHV, also referred to as human herpesvirus 8 [HHV8]) infected cells [2–5]. Infection with KSHV is commonly associated with fatal malignancies, is the causative agent of primary effusion lymphoma (PEL) and Kaposi’s sarcoma (KS) and is frequently associated with multicentric Castleman’s disease (MCD) [6,7]. Like all herpesviruses, KSHV infection is lifelong and has two distinct phases to its lifecycle; latent and lytic. During latency, viral gene expression is highly restricted and, in the tumor setting, involves the expression of the latency associated nuclear antigen (LANA), the viral FLICE inhibitory protein (vFLIP), viral cyclin, kaposin and various virally encoded miRNAs. Together these promote tumorigenesis in all known KSHV-associated malignancies. Nevertheless, at least for KS, the lytic phase of KSHV, which results in the expression of the complete viral genome and the production of infectious virions, is necessary for sarcomagenesis. For this reason, the molecular mechanisms governing the switch from latency to lytic reactivation have received much attention as they may provide excellent targets for therapeutic intervention.

Current treatments of KSHV-associated malignancies have limited efficacy. PEL is treated using a combination of cyclophosphamide, doxorubicin, vincristine and prednisone (similar to CHOP therapy) and/or highly active retroviral therapy (HAART) [8,9]. For AIDS-related KS, HAART is also favored, and due to the requirement of KSHV lytic infection for the pathogenesis of KS, anti-herpesviral drugs have also been used [10]. More recently, preclinical models have demonstrated that inhibition of the UPS using bortezamib [2–5], or bortezamib in combination with a histone deacetylase (HDAC) inhibitor (vorinostat) may provide a promising new avenue [11]. Given the success of bortezamib (marketed as Velcade) for the treatment of multiple myeloma and mantle cell lymphoma, there are now various additional small molecule inhibitors of the UPS at various stages of development [1], as well as drugs that target ubiquitin-like pathways such as the NEDDylation cascade [12].

The attachment of ubiquitin-like (Ubl) proteins to substrates represents one of the most vital posttranslational modifications in the cell and, so far, twelve Ubls have been identified including ubiquitin (Ub), NEDD8 and SUMO. The covalent attachment of Ubls to substrates requires an enzyme cascade involving an E1 activating enzyme, an E2 conjugating enzyme and an E3 ligase that provides substrate specificity, all of which are potential ‘drugable’ targets. Ubiquitylation is the principal mechanism by which proteins are targeted for proteasomal degradation, and the largest class of E3 ligases are the Cullin-RING ligases (CRLs) [13]. These modular proteins are centered on one of several Cullin scaffolds (Cul1, 2, 3, 4A, 4B, 5 and 7) and consist of a RING-binding protein (RBX1 or RBX2) that interacts with the Ub-loaded E2 and a substrate adapter protein that brings the target into close proximity with the Ub-E2. Many substrate adapters have been identified, and the various combinations of Cul-adapter complexes means there are several hundred possible CRLs, with several-fold more substrates [14]. Intriguingly, the activity of CRLs is dependent on themselves being modified by the Ubl NEDD8, which requires the activity of the NEDD8 activating enzyme, NAE1 [15]. Recently, a specific NAE1 inhibitor has been developed (MLN4924) and is currently in clinical trials for various malignancies. MLN4924-mediated inhibition of NEDDylation blocks CRL activity resulting in the global stabilization of CRL targets [12].

While the ubiquitin and SUMO pathways have been extensively studied in infectious diseases [16,17], much less attention has been given to the role of NEDD8. Recent work has shown that the large tegument protein of gammaherpesviruses has deNEDDylase activity and this was important for EBV infection [18]. Another study demonstrated that inhibition of NEDDylation blocked the ability of HIV to degrade the cellular restriction factor APOBEC3G via Vif-mediated hijack of Cul5 [19]. There are indications in the literature that NEDDylation is likely to play a central role in viral pathogenesis. For example, HIV is known to modulate the activity of at least three cullins [20,21], the KSHV LANA protein forms a CRL complex with Cul5 and targets p53 [22], and it was recently shown that human cytomegalovirus (HCMV) utilizes the nucleotide excision repair (NER) pathway, which is regulated predominantly by Cul4A/B [23], to repair its genomes during viral replication [24].

Here we asked if NEDDylation is important for the KSHV lifecycle, and to begin to dissect the functional consequences of its inhibition. Treatment of cancer cells with MLN4924 leads to dramatic cytotoxicity, and some of the best characterized mechanisms include the induction of DNA re-replication by blocking the degradation of Cdt-1 [25,26], or by inhibiting NF-κB signaling via stabilization of the inhibitor of NF-κB protein IκBα [27], both of which eventually lead to apoptosis. Given the essential role of NF-κB for the maintenance of viral latency and the survival of PEL [28], we reasoned that these cells would be highly sensitive to MLN4924. We found that, indeed, MLN4924 led to significant PEL cytotoxicity and this was mediated via inhibition of NF-κB signaling. Intriguingly, we also showed that NEDDylation was essential for amplification of the KSHV genome during reactivation of the lytic cycle and that treatment with MLN4924 prevented the recruitment of RTA to the origin of lytic replication (OriLyt). This work has highlighted the essential role of NEDD8 and CRL-mediated ubiquitylation during the life cycle of KSHV and suggests that NEDDylation may provide a novel therapeutic target for the treatment of KSHV-associated malignancies.

## Results

### Inhibition of NEDDylation is cytotoxic to PEL cells and leads to lytic cycle-associated gene expression

**Fig 1:**
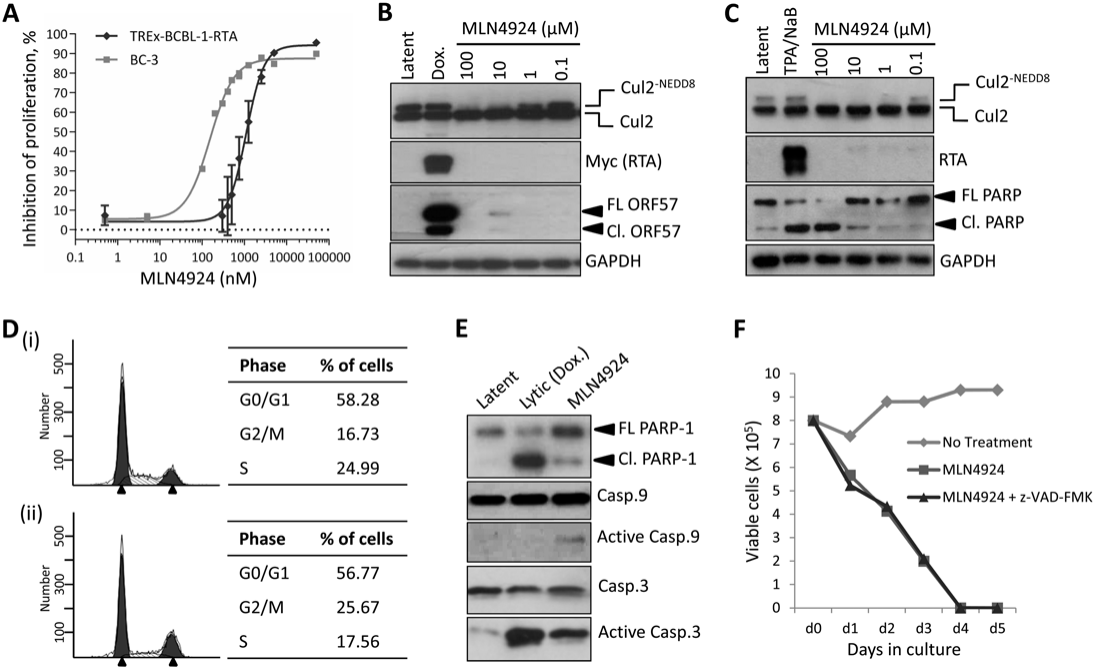
Inhibition of NEDDylation is cytotoxic in PEL cells. (**A**) TREx-BCBL-1-RTA and BC-3 cells were plated into 96-well plates and treated at varying concentrations of MLN4924. Cells viability was determined 96 h later using an ATPlite assay, and shown is a representative trace of several experiments, and EC50s were calculated (TREx-BCBL-1-RTA = 1.10 μM; BC-3 = 0.15 μM). (**B**) Immunoblot analysis of TREx-BCBL-1-RTA cells showing that MLN4924 effectively inhibits NEDDylation in PEL cells as shown by reductions in NEDDylated Cul2 after 24 h treatment. Also, treatment appeared to reactivate KSHV lytic gene expression (see ORF57 expression in 10 μM lane). (**C**) Similar to (B), but with a second PEL cell line, BC-3. As for TREx-BCBL-1-RTA, treatment led to the activation of lytic gene expression as shown by endogenous RTA expression. Cleavage of PARP also indicated that MLN4924 activates apoptosis in this cell line. (**D**) Cell cycle analysis of TREx-BCBL-1-RTA cells treated for 24 h with 1 μM MLN4924 showing a reduction in S-phase and an accumulation of cells in G2/M. (**E**) TREx-BCBL-1-RTA cells treated with 10 μM MLN4924 for 24 h activated caspase 3/7 as indicated by PARP-1 cleavage. In contrast to lytic reactivation by Dox., MLN4924 led to the activation of initiator Caspase 9 which presumably led to Caspase 3 cleavage (activation). (**F**) Stationary cultures of BCBL-1 cells were treated with MLN4924 and cell viability was monitored by trypan blue exclusion over 5 days. Cells were pre-treated with z-VAD-FMK just prior to treatment to inhibit caspase-mediated apoptosis; however, this did not prevent cytotoxicity. FL PARP-1 denotes full length PARP-1; Cl. PARP-1 denotes cleaved PARP-1. FL ORF57 denotes full length ORF57 and Cl. ORF57 denotes the caspase 7-cleaved ORF57 [29].

Firstly, it was important to confirm that NEDDylation still occurred in KSHV-infected cells. Transfection of rKSHV-219 cells with FLAG-NEDD8 and the indicated Myc-tagged Cullin, followed by immunoprecipitation of NEDD8 and immunoblot analysis confirmed that Cullin proteins were modified in infected cells (S1 Fig). As MLN4924 has demonstrated anti-proliferative activity in various cancer cell lines, we assessed its cytotoxic effects in PEL cells. We treated PEL cells (BCBL-1, TREx-BCBL-1-RTA and BC-3) with varying concentrations of MLN4924 and determined cell viability after 96 h using a luminescence ATP detection assay. These data demonstrated that MLN4924 was indeed cytotoxic to latently infected PEL cells with approximate EC_50_ values of 1.10 μM and 0.15 μM, for TREx-BCBL-1-RTA and BC-3 respectively (Fig. 1A). However, it was interesting to note that a low level of ORF57 expression was observed in TREx-BCBL-1-RTA (see 10 μM lane) and low levels of endogenous RTA in BC-3 cells treated with MLN4924, suggesting that drug treatment alone induced lytic gene expression (Fig. 1B & C). We also noted that MLN4924 led to apoptosis in BC-3 cells as shown by the cleavage of PARP (Fig. 1C). To investigate MLN4924-induced cytotoxicity, cell cycle analysis of TREx-BCBL-1-RTA cells was performed 24 h after treatment with 1 μM MLN4924. Compared to untreated TREX-BCBL-1-RTA cells (Fig. 1D (i)), MLN4924 treatment led to a reduction in S-phase and an accumulation of cells in G2/M (Fig. 1D (ii)). Clearly, inhibition of NEDDylation was toxic to PEL cells, and as shown for BC-3 this led to apoptosis. To further investigate this, we tested the activation of caspases in TREx-BCBL-1-RTA cells. Similar to BC-3, treatment of TREx-BCBL1-RTA led to the cleavage of PARP, indicative of Caspase 3/7 activation, as did the reactivation of the KSHV lytic cycle after the addition of Dox. (Fig. 1E). It has been shown previously that induction of the lytic cycle activates initiator Caspase 8, but not Caspase 9 in order to activate effector caspases such as Caspase 3 and 7 [29]. In agreement with this, we also showed that lytic reactivation did not activate Caspase 9; nevertheless, MLN4924 did, resulting in the activation of Caspase 3 (Fig. 1E). Importantly however, caspase inhibition (using the pan-caspase inhibitor, z-VAD-FMK) did not prevent PEL cell death after treatment with MLN4924 (Fig. 1F). This result showed that PEL cytotoxicity was not solely a result of apoptosis and suggests other factors, such as the inhibition latency-associated gene expression may contribute to cell death.

**Fig 2:**
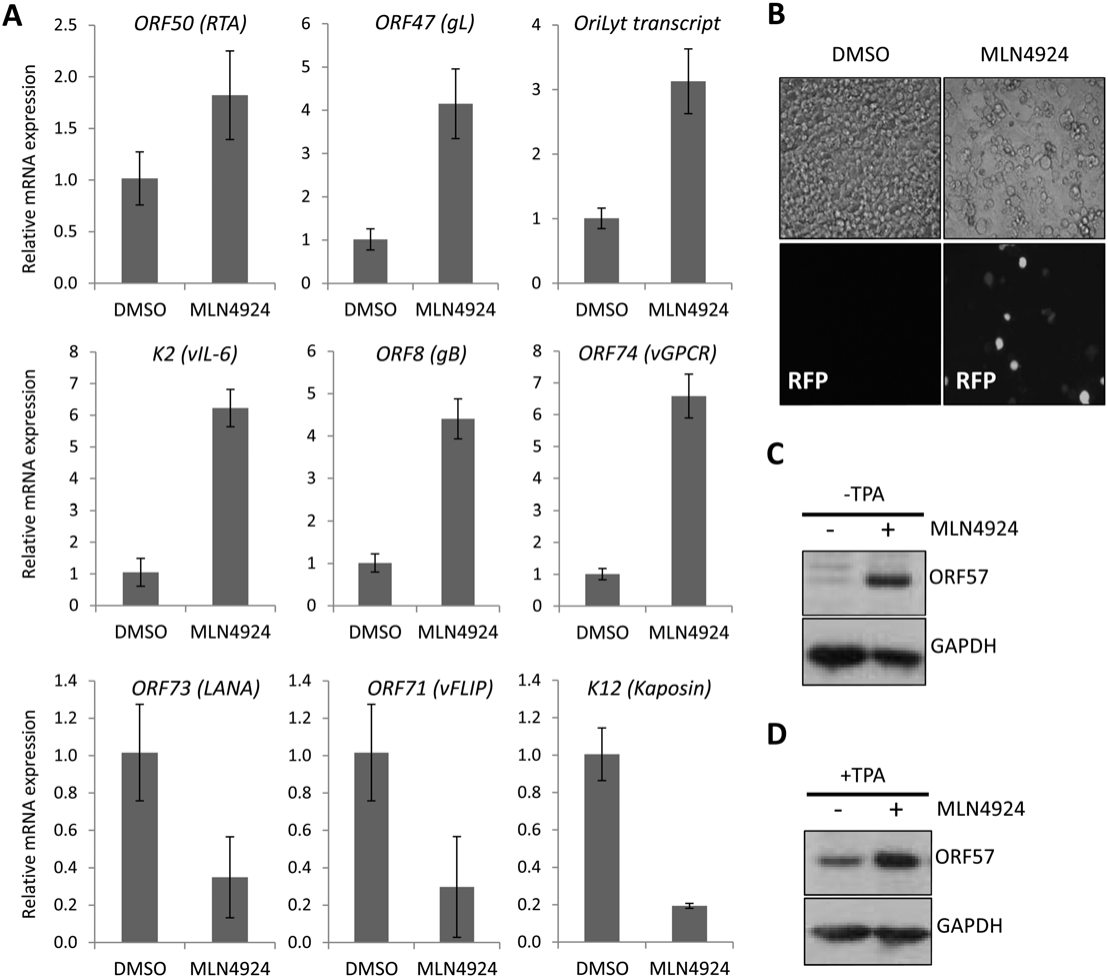
MLN4924 induced lytic cycle—associated expression. (**A**) Latently infected TREx-BCBL-1-RTA cells were treated with 1 μM MLN4924 and total RNA was isolated from samples harvested 24 h later. The normalized expression (using *GAPDH* expression) of lytic cycle genes and latency associated (*ORF73*, *ORF71 and K12*) genes were analyzed by qRT-PCR and compared to the levels found in DMSO-treated control samples. Three biological replicates were independently analyzed and the error bars represent the standard deviation from the mean. (**B**) The inhibition of NEDDylation leads to reactivation of lytic cycle protein expression; rKSHV.219 cells were treated with 0.1 μM MLN4924 (a concentration that was tolerated for the duration of the experiment) for 36 h. Red fluorescent protein (RFP) expression was observed in numerous MLN4924-treated cells, indicative of RTA protein expression. RFP expression was observed in a minority DMSO-only treated cells (as expected due to low level spontaneous reactivation). (**C**) ORF57 expression was also detected by immunoblot analysis of MLN4924-treated rKSHV.219 cells. (**D**) Inhibition of NEDDylation enhances reactivation. MLN4924-treated rKSHV.219 cells were incubated with or without TPA (able to reactivate KSHV) for 36 h.

Therefore, we next measured the effect of treatment on viral gene expression. Latently infected TREx-BCBL-1-RTA were treated with 1 μM MLN4924 and harvested 24 h later. As shown in Fig. 2A, the expression of all lytic-cycle associated genes tested were induced after inhibition of NEDDylation. In contrast, the expression of latency-associated transcripts *ORF73* (latency associated nuclear antigen [LANA]) and *ORF71* (vFLIP) were reduced in the presence of drug. Interestingly, the expression of *K12* followed that of latency-associated genes. While K12 is dramatically induced upon activation of the KSHV lytic cycle [30], its expression during latency is initiated from the LANA promoter [31]. This suggested that MLN4924 treatment modulated latency-associated viral gene expression, rather than inducing the full KSHV lytic cycle. To further investigate the potential of MLN4924 to reactivate lytic cycle gene expression, rKSHV.219 cells were cultured for 36 h in the presence of 0.1 μM MLN4924, a concentration that was tolerated while in culture. These HEK293-based cells are latently infected with recombinant KSHV that contains a red fluorescent protein (RFP) reporter gene under the control of an RTA-responsive promoter (the *PAN* promoter); hence expression of RTA (and thus reactivation of the lytic cycle) leads to RFP expression. As shown in Fig. 2B, RFP expression was observed in numerous cells demonstrating that MLN4924 was indeed able to induce the expression of RTA. MLN4924 was also capable of initiating expression of ORF57 (activated by RTA during reactivation) (Fig. 2C) and enhancing lytic cycle gene expression in cells reactivated with TPA alone (Fig. 2D). Taken together, these results confirmed that inhibition of NEDDylation was clearly essential for PEL viability and that its inhibition was able to induce low level, lytic cycle-associated viral gene expression. We next investigated the potential mechanisms of MLN4924-mediated cytotoxicity.

### MLN4924-induced cytotoxicity is due to the inhibition of NF-κB

The reported mechanisms of MLN4924-induced cytotoxicity is the induction of DNA re-replication (by blocking the CRL-mediated degradation of Cdt-1) [26], or by inhibiting NF-κB signaling as CRL-mediated degradation of IκBα is prevented, thus precluding NF-κB’s transcriptional activity [27]. It is well established that PEL is dependent on NF-κB signaling, and that KSHV vFLIP drives the constitutive activation of NF-κB in order to maintain viability. NF-κB activity also inhibits *RTA*-mediated transactivation of lytic genes, and thus it is important for maintaining latency. As the viral gene expression profiles following MLN4924 treatment (Fig. 2A) are consistent with an inhibition of NF-κB, we tested whether it was inhibited after MLN4924 treatment. The NF-κB transcription factor is tightly regulated via its interaction with IκBα which maintains it in the cytoplasm and away from its transcriptional targets. Upon stimulation, the Iκκ complex phosphorylates IκBα at ser32 and 36, which signals for the recruitment of the Cul1-containing βTrCP E3 ligase leading to the subsequent degradation of IκBα. This releases NF-κB allowing to translocate to the nucleus and drive transcription [32]. Therefore, MLN4924 inhibition of CRL1 function should stabilize phosphorylated IκBα (pIκBα) leading to an inhibition in NF-κB function.

**Fig 3:**
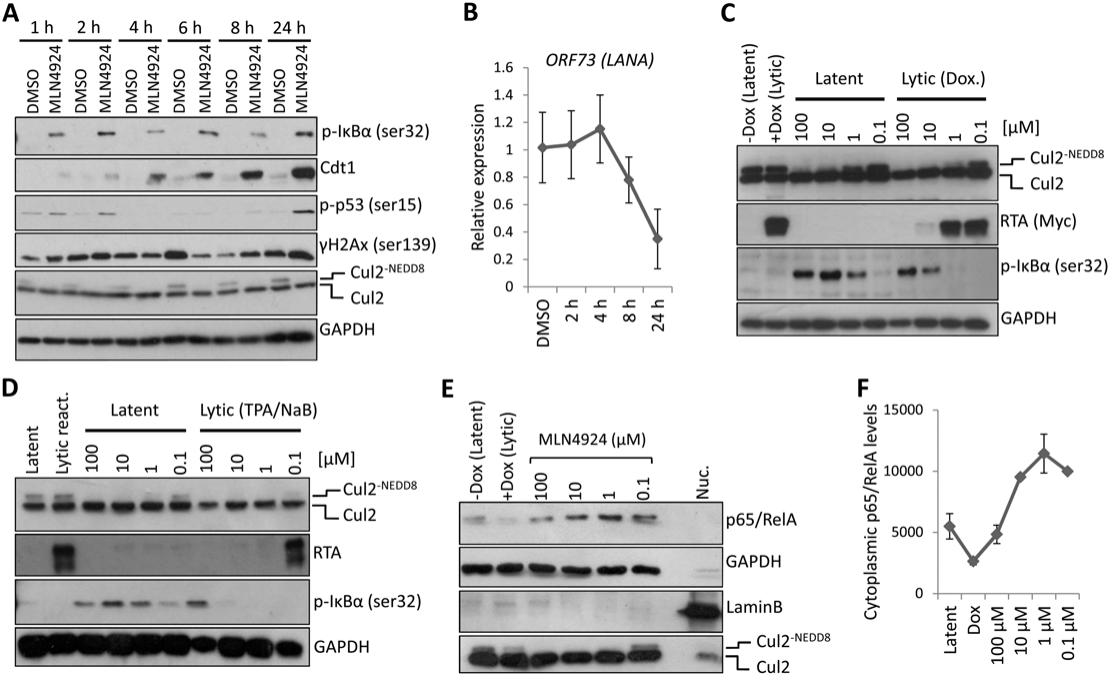
PEL cytotoxicity is due to NF-κB inhibition. (**A**) TREx-BCBL-1-RTA cells were treated with 5 μM MLN4924 for the indicated times. Cell lysates were analyzed by immunoblot using the indicated antibodies to determine the mode of cytotoxicity. (**B**) Transcription of NF-κB-regulated latency-associated gene *LANA* was determined at 2, 4, 6 and 8 h post treatment with 5 μM MLN4924 to investigate the point at which the inhibition of NEDDylation had an effect. (**C**) MLN4924 treatment led to the stabilization of phosphorylated IκBα (pIκBα[ser32]) in TREx-BCBL-1-RTA cells, IκBα expression is reduced in lytic cells reactivated by the addition of Dox. (**D**) Similar to (C), pIκBα is stabilized in MLN4924-treated BC-3 cells. (**E**) Cytoplasmic fractions were enriched (as shown by the lack of nuclear Lamin B and enrichment of GAPDH—compare to nuclear fraction [Nuc.]) from TREx-BCBL-1-RTA cells treated with varying concentrations of MLN4924 which shows the stabilization of the p65/RelA subunit of NF-κB in the cytoplasm. (**F**) Densitometry analysis using ImageJ of the immunoblot analysis shown in (E).

To investigate the most likely mechanism of MLN4924-induced cytotoxicity we performed a timecourse experiment (Fig. 3A). Here we show that as little as 1 h treatment of TREX-BCBL-1-RTA cells with MLN4924 led to an inhibition of NEDDylation (as shown by the lack of NEDDylated Cul2 compared to DMSO-treated cells). We also observed the stabilization of pIκBα as early as 1 h post-treatment. By contrast, the accumulation of Cdt1 did not occur until 4 h with significant levels not observed until 6–8 h later. Furthermore, cell cycle analysis of TREx-BCBL-1-RTA cells revealed an accumulation of cells in G2/M after 24 h, indicative of a block at the DNA damage check point. Concomitant with this, we observed the phosphorylation of p53 at 24 h post treatment, but not at earlier time points. Likewise, we did not observe significant alterations in the levels of phosphorylated H2Ax (γH2Ax)—a marker of DNA strand breaks—although γH2Ax is known to be present in latently infected cells due to its association with LANA expression [33]. Importantly, the MLN4924-associated reduction of *ORF73* mRNA expression occurs prior to any indication of DNA damage (Fig. 3B). Along with cell cycle analysis (Fig. 1D) showing that treatment of TREx-BCBL-1-RTA cells led to a reduction of cells in S-Phase (which is inconsistent with Cdt1-associated DNA-rereplication), and the rapid stabilization of pIκBα suggests that inhibition of NF-κB signaling was responsible for cytotoxicity. Additionally, pIκBα levels increased in an MLN4924-dose-dependent manner in latently-infected TREx-BCBL-1-RTA (Fig. 3C) and BC-3 cells (Fig. 3D) treated for 24 h, showing that, as expected, treatment did stabilize pIκBα. In accordance with this, the cytoplasmic levels of the p65/RelA subunit of NF-κB were increased in a dose-dependent manner (Fig. 3E-F—enrichment of the cytoplasmic compartment was verified by the lack of nuclear Lamin B—compared to the nuclear fraction control which maintained Lamin B but lacked GAPDH). Previous reports have shown that upon lytic reactivation, *IκBα* is posttranscriptionally downregulated [34] and consistent with this, cytoplasmic p65/RelA decreased in Dox-treated (reactivated) cells further highlighting the involvement of IκBα for MLN4924-induced inhibition of NF-κB.

Additionally, we did not observe pIκBα expression in reactivated cells (those with RTA expression) regardless of MLN4924 treatment (Fig. 3C-D). This was also highlighted in our confocal analysis of p65/RelA localization showing it had translocated to the nucleus in reactivated cells even when treated with MLN4924 (S2 Fig). We therefore surmised that inhibition of NF-κB, via stabilization of IκBα was responsible for cytotoxicity and demonstrated that inhibition of NEDDylation may provide a therapeutic option for the treatment of NF-κB-dependent PEL.

### Identification of Cul4B as a novel regulator of KSHV latency

**Fig 4:**
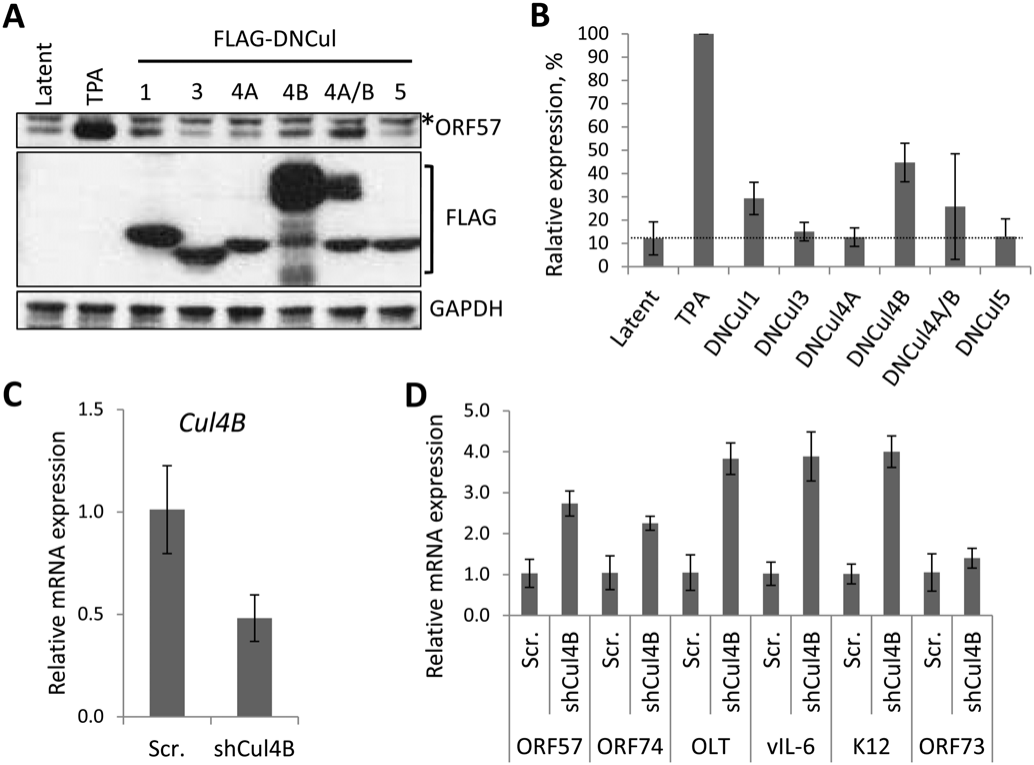
The role of individual CRLs for the regulation of KSHV latency. (**A**) Dominant-negative versions of each Cullin (FLAG-DNCul that inhibits CRL activity [35]) was expressed in latently infected rKSHV.219 cells and ORF57 expression (as a marker of lytic reactivation) was tested by immunoblot analysis 36 h later. Treatment of cells with MLN4924 and TPA/NaB (sodium butyrate) served as positive controls for reactivation. *denotes non-specific bands and error bars represent the standard deviation of the mean of two independent transfection experiments. (**B**) Densitometry analysis of (A). (**C**) qRT-PCR analysis of *Cul4B* mRNA from TREx-BCBL-1-RTA cells transfected with either a scramble control shRNA expression vector (Scr.) or four independent shRNA expression vectors targeted at *Cul4B* (shCul4B). RNA was harvested four days post transfection. (**D**) qRT-PCR analysis of viral gene expression in Cul4B-knockdown cells normalized against *GAPDH* expression. Error bars represent the standard deviation from three independent transfections per condition. OLT denotes OriLyt transcript.

Treatment of cells with MLN4924 blocks total NEDDylation, which undoubtedly has far-reaching implications on cellular function. To determine if inhibition of CRL activity was responsible for the phenotypes we have observed, and to identify which CRLs are implicated, we expressed dominant-negative versions of the different cullin family members (DNCul) in cells latently infected with KSHV (rKSHV.219) and screened for reactivation of the lytic cycle by expression of ORF57. DNCuls are truncated forms of cullin proteins that are still able to engage their respective substrates, but are unable to bind to the Ub-loaded E2 enzyme (and are unable to be NEDDylated), thus preventing substrate degradation [35]. Using this approach, we showed that CRL1 was able to reactivate ORF57 expression, albeit at a low level (Fig. 4A-B). That CRL1 inhibition reactivated lytic expression is not surprising as it is known to regulate NF-κB signaling, corroborating our earlier analyses (Fig. 2 & 3) [36,37]. However, we were surprised to note that CRL4B inhibition was able to reactivate ORF57 expression (Fig. 4A-B). Cul4A and 4B both regulate chromatin-associated functions, and they share ca. 80% amino acid identity [38]. However, Cul4B has an extended N-terminus that contains a nuclear localization signal (NLS) and its expression is confined to the nucleus, whereas Cul4A is recruited to the nucleus following its interaction with DDB1 in response to genotoxic stress. Due to the high degree of similarity, they are both capable of binding the same CRL4 components (e.g. DNA damage binding proteins 1 and 2 [DDB1, DDB2] and the various DDB and Cul4-associated factors [DCAFs]). Interestingly, when DNCul4A and 4B were expressed together, the activation of ORF57 was reduced. We believe that this was due to the fact that these two proteins compete for the same binding partners meaning that the degree of nuclear inhibition of CRL4B was reduced, further implicating the importance of CRL4B in the regulation of KSHV latency. Moreover, as CRL4B resided in the nucleus, this particular E3 ligase is the most likely one to be involved in KSHV biology. To confirm this, independent shRNA knockdown of Cul4B in TREx-BCBL-1-RTA cells was performed which led to an ca. 50% reduction in *Cul4B* expression (Fig. 4C) resulting in the expression of various lytic cycle-associated genes (Fig. 4D). Interestingly, expression of the latency-associated gene *LANA* did not alter suggesting that Cul4B specifically regulated the expression of lytic genes. Here, we also investigated the expression of *K12*, which in contrast to the reduced expression we observed in the presence of MLN4924, was increased by Cul4B knockdown (Fig. 4D). Although a thorough investigation of individual CRLs is required (for example, using RNAi experiments to inhibit the various CRL components), this data suggests that MLN4924’s effect on KSHV latency is through its inhibition of CRL activity. In addition, we have identified CRL4B as a novel regulator of KSHV latency.

### NEDDylation is essential for replication of the KSHV genome

**Fig 5:**
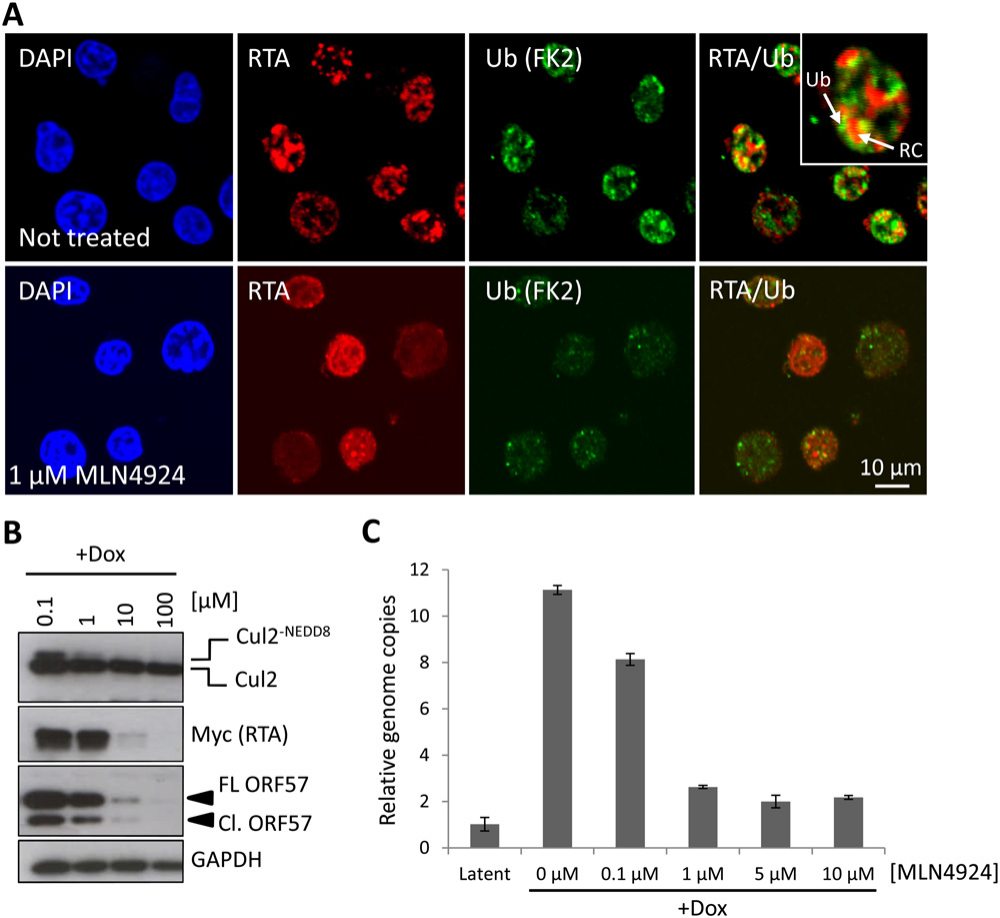
NEDDylation is required for lytic reactivation. (**A**) To test if NEDDylation-associated ubiquitylation is a feature of KSHV lytic reactivation, immunofluorescence analysis was used. TREx-BCBL-1-RTA cells were left untreated, or treated with MLN4924 and reactivated by the addition of Dox. Soluble nuclear proteins (i.e. those not associated with chromatin) were removed prior to fixation (see Materials and Methods). Ubiquitylated proteins (using antibodies specific for ubiquitylated proteins and not free ubiquitin) were seen associated with the edges of KSHV replication compartments (inset; RC denotes replication compartment; Ub denotes ubiquitin-modified proteins), as indicated by RTA staining in untreated, but dox. reactivated cells and the level of ubiquitylation was reduced in the presence of 1 μM MLN4924. (**B**) Cell lysates were prepared from TREx-BCBL-1-RTA cells that were treated with MLN4924 and reactivated with Dox for 24 h. Immunoblot analysis of viral proteins shows that lytic-associated protein expression is inhibited in a dose-dependent manner (**C**) TREx-BCBL-1-RTA cells were treated at varying concentrations of MLN4924, reactivated with doxycycline and total DNA was isolated 72 h later. Normalized (to cellular GAPDH) viral genome replication was measured using qPCR analysis of three biological replicates; error bars represent standard deviation from the mean.

The KSHV lytic cycle is intimately linked to the pathogenesis of KSHV malignancies and the role of NEDDylation or the importance of CRL-mediated ubiquitylation during this process is currently unknown. To investigate if ubiquitylation is indeed a feature of KSHV reactivation, we stained reactivated cells with anti-Ub FK2 (which only recognizes ubiquitylated proteins and not free ubiquitin) and to increase the stringency of this assay, we removed soluble nuclear material prior to fixation (see Materials and Methods). As shown in Fig. 5A, reactivation is associated with significant levels of ubiquitylation which appeared to localize to the edges of the replication compartments (Fig. 5A inset). However, in the presence of low concentration MLN4924 (1 μM), the degree of replication compartment ubiquitylation was reduced. There are two potential reasons that explain this observation; that CRLs are the predominant E3 ligases that ubiquitylate replication compartment factors, or that NEDDylation is required within replication compartments, and this is important for recruiting ubiquitin E3 ligases (as recently demonstrated during the DNA double-strand break repair mechanism [39]). As NEDDylation-dependent ubiquitylation occurred around replication compartments, we asked whether this modification was required for KSHV lytic reactivation. For these experiments, we employed TREx-BCBL-1-RTA cells, where the lytic cycle is induced by the addition of doxycycline (Dox.) by virtue of Dox-inducible expression of exogenous RTA-Myc. The advantage of using TREx-BCBL-1-RTA cells is that the lytic cycle is robustly induced (more so than with TPA/NaB) and any effect is a result of viral gene expression and not due to the general effects of TPA/NaB. Cells were treated with varying concentrations of MLN4924, the lytic cycle was induced and 24 h later cells were harvested and viral protein expression was assessed by immunoblotting. As shown in Fig. 5B, MLN4924 was able to inhibit the expression of RTA-Myc and its downstream target ORF57 in a dose-responsive manner, corresponding to the levels of Cul2 NEDDylation. As RTA-Myc represents exogenous protein expression (i.e. not from the KSHV genome) in TREx-BCBL-1-RTA cells, these results showed that MLN4924 inhibited all transcription in these cells. Using qPCR analysis of viral genomes, we also confirmed that MLN4924 was able to inhibit KSHV DNA replication (Fig. 5C). However, KSHV genome replication was inhibited at lower concentrations than was required to inhibit viral protein expression (compare protein expression and genome replication levels in cells treated with 1 μM) highlighting a potentially novel mechanism, mediated by NEDDylation, that uncouples these two features of KSHV biology. These data show for the first time that NEDDylation (or NEDDylation-dependent ubiquitylation) plays a significant role during the KSHV lytic cycle.

### MLN4924-mediated inhibition of KSHV genome replication is not due to caspase activation

**Fig 6:**
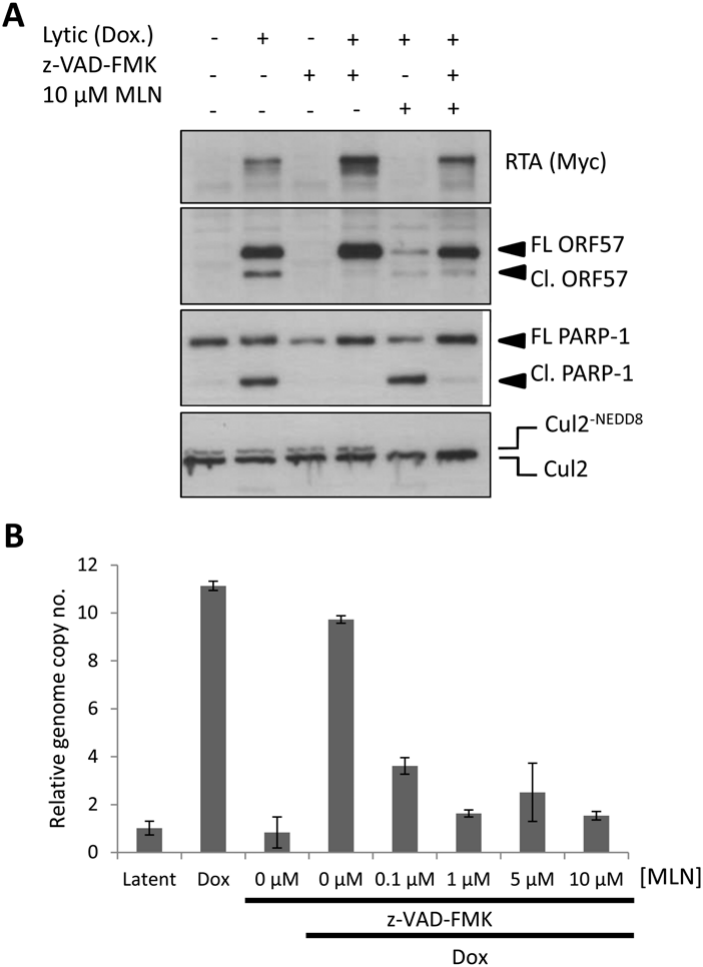
Inhibiting MLN4924-induced apoptosis does not restore lytic reactivation. (**A**) Inhibition of caspase activity (using the pan-caspase inhibitor z-VAD-FMK) restored lytic cycle protein expression in reactivated cells treated with MLN4924. Successful inhibition of caspase activity was monitored by immunoblot analysis of PARP-1 and ORF57 cleavage. FL ORF57 denotes full length ORF57; Cl. ORF57 denotes cleaved ORF57. (**B**) Despite restoration of protein expression upon caspase inhibition, viral genome replication was still blocked. TREx-BCBL-1-RTA cells were treated as indicated and cells were harvested 72 h later. Total DNA was harvested and qPCR was used to determine KSHV genome copies relative to latent cells (as in Fig. 5C).

We have shown that the inhibition of NEDDylation leads to caspase activation (Fig. 3B) which may account for the ability of MLN4924 to prevent KSHV genome replication. Furthermore, caspase activation has been shown to regulate viral gene expression via Caspase 7 cleavage of ORF57 [29]. We therefore asked whether caspases were responsible for the inhibition of KSHV reactivation. Treatment of cells with z-VAD-FMK efficiently blocked caspase-mediated cleavage of PARP-1 (Cl. PARP-1) and ORF57 (Cl. ORF57) in reactivated cells; caspase inhibition also led to an increase in viral protein expression as noted by the increased levels of full length ORF57 (FL ORF57) and RTA expression in reactivated cells (lane 4, Fig. 6A). Importantly, inhibition of caspase activity restored ORF57 and RTA-Myc expression, clearly demonstrating that MLN4924-induced apoptosis was responsible for inhibiting viral protein expression (lane 6, Fig. 6A). To our surprise however, when we treated cells with z-VAD-FMK and MLN4924 and investigated viral genome copy number by qPCR, viral DNA replication was still inhibited (Fig. 6B).

**Fig 7:**
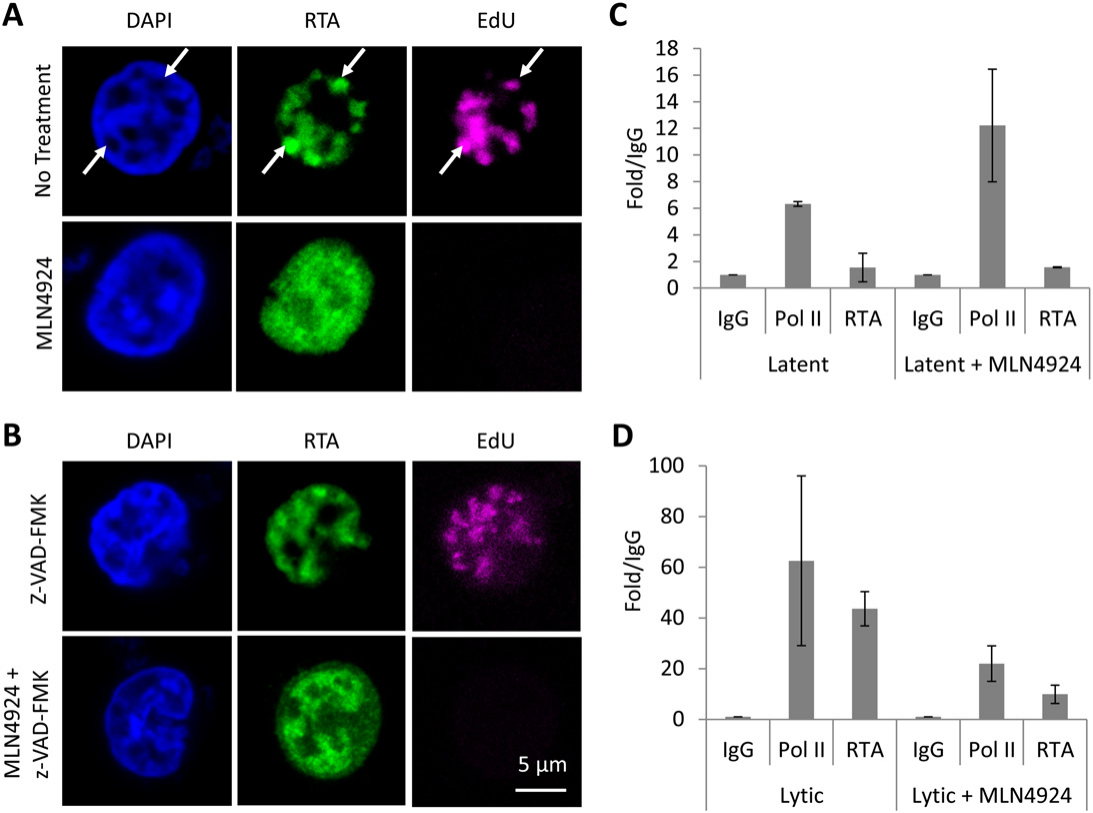
MLN4924 prevents the proper organization of replication compartments and the recruitment of RTA to OriLyt. (**A**) Replication compartments were observed in TREx-BCBL-1-RTA cells reactivated by Dox. for 16 h by imaging for the incorporation of EdU into replicating KSHV DNA, and its colocalization with RTA (arrows, top panel). Treatment with MLN4924 inhibited the formation of replication compartments (as observed by pan-nuclear staining of RTA, bottom panels) and blocked the replication of viral DNA (as indicated by the absence of EdU in RTA-positive cells). (**B**) Reactivated cells treated with caspase inhibitor z-VAD-FMK still formed replication compartments, yet z-VAD-FMK was unable to restore KSHV DNA replication in MLN4924 treated cells. (**C**) Latent TREx-BCBL-1-RTA cells were treated with 1 μM MLN4924 for 18 h and ChIP analysis was performed using antibodies specific for IgG (isotype control), RNA Pol II and Myc-tag (for RTA), and primers specific for the RTA responsive element (RRE) of OriLyt. Treatment induced RNA Pol II recruitment (with a slight increase in RTA) corroborating our transcriptional analyses (see Fig. 2). (**D**) The same analysis was performed but in cells reactivated with Dox. This analysis showed that MLN4924 treatment blocked the recruitment of RTA onto OriLyt during lytic reactivation. Error bars represent the standard deviation of the mean from two independent ChIP experiments.

An additional measure of viral genome replication involves the incorporation of EdU into DNA specifically marking replicating viral genomes in replication compartments, where proteins such as RTA are also localized (Fig. 7A). However, even in cells clearly expressing RTA, MLN4924 treatment prevented virus replication, as shown by the lack of EdU-positive replication compartments. Inhibition of caspases restored viral protein expression in MLN4924-treated cells; however, viral DNA replication was still inhibited (Fig. 6B). We therefore repeated our EdU analysis of drug-treated cells in the presence of z-VAD-FMK. Inhibition of caspases permitted the formation of replication centers and amplification of KSHV DNA in non-treated cells. Nevertheless, z-VAD-FMK was still unable to restore viral genome replication in MLN4924-treated cells (Fig. 7B). These data confirm that MLN4924-mediated activation of caspases was not responsible for the inhibition of KSHV genome replication upon MLN4924 treatment.

### NEDDylation is required for the proper formation of KSHV replication compartments and the recruitment of RTA to OriLyt

Throughout our studies, we noticed that reactivated cells treated with MLN4924 displayed an unusual RTA expression pattern; as shown in Figs. 5A & 7A, MLN4924 treatment is associated with pan-RTA localization, rather than in discrete, EdU-positive foci. We also observed the same phenotype when we costained for RTA and a second replication compartment-associated factor, RNA Pol II (S3 Fig). We therefore hypothesized that this would have implications for RTA’s ability to mediate KSHV genome replication, and therefore provide a rationale for the MLN4924-induced block in replication. OriLyt is the *cis*-acting loci where proteins required for KSHV genome replication assemble. The mechanisms of this have been well characterized and RTA is central to this process. For example, it has been shown that the pre-replication complex (which involves RTA, K8, the core replication proteins and various cellular proteins) is formed prior to its loading onto OriLyt, and that RTA, through its direct DNA binding activity with RRE, recruits these factors to OriLyt [40–42]. Therefore, as MLN4924 inhibited viral genome replication and prevented the correct localization of RTA, we asked whether treatment precluded the loading of the replication complex. To do this, TREx-BCBL-1-RTA cells were treated with 1 μM MLN4924 (a concentration that permits viral protein expression but inhibits replication) and 18 h later, ChIP analysis was performed using antibodies specific for IgG (isotype control), RNA Pol II and Myc-tag (for RTA), and primers specific for the RTA responsive element (RRE) of OriLyt. Firstly, we performed this analysis on latently infected cells (Fig. 7C), where we observed an enrichment of RNA Pol II (6.45-fold over IgG), whereas RTA levels were lower than those observed for IgG control. Interestingly, when cells were treated, RNA Pol II occupancy at OriLyt approximately doubled, and RTA levels increased 1.6-fold over IgG. These results confirm that MLN4924 was able to induce transcription in latently infected cells; however, they show that only low levels of RTA are required. During lytic replication, both RNA Pol II (63-fold over IgG) and RTA (44-fold over IgG) were increased at OriLyt (Fig. 7D). However, after MLN4924 treatment, RNA Pol II occupancy reduced ca. 3-fold (to 21-fold over IgG) and RTA occupancy decreased ca. 4-fold (to 10-fold over IgG) confirming that the inhibition in KSHV genome replication was due to a block in the recruitment of the viral pre-replication complex. Consequently, by using MLN4924 to investigate the role of NEDDylation in KSHV biology, we have uncovered a novel mechanism that regulates lytic reactivation.

## Discussion

KSHV infection is responsible for various malignancies, including KS, PEL and many cases of MCD. As these diseases are highly associated with compromised immune function, they represent some of the most common cancers in areas of the world where HIV infection is also high. Indeed, KS is the most prevalent cancer in many sub-Saharan countries. Therefore, understanding the molecular mechanisms that underlie KSHV biology is of the utmost importance if therapeutic targets are to be identified. Given the success of drugs such as bortezamib (UPS inhibitor) for the treatment of various malignancies, interest has grown in the development of inhibitors that target additional aspects of the UPS, or additional Ubls. An example of this is MLN4924, a small molecule inhibitor that blocks the function of NAE1, the first enzyme (E1) in the NEDDylation cascade. Inhibition of NEDDylation leads to a global stabilization of CRL targets, and this drug has proved to be potently cytotoxic in many cancer models. The principal mechanisms of MLN4924 cytotoxicity in cancer cells appear to involve blocking NF-κB signaling (via stabilization of IκBα, and the retention of the NF-κB transcription factor in the cytoplasm) or leading to unlicensed DNA replication (via stabilization Cdt-1), ultimately leading to apoptotic cells death. Given that PEL cells absolutely require NF-κB signaling for their survival, we asked whether MLN4924 was able to kill these cells. Furthermore, as the lytic cycle plays a significant role during the pathogenesis of KS, we also asked whether the NEDDylation cascade was necessary for virus reactivation, and if so, what role it plays.

**Fig 8:**
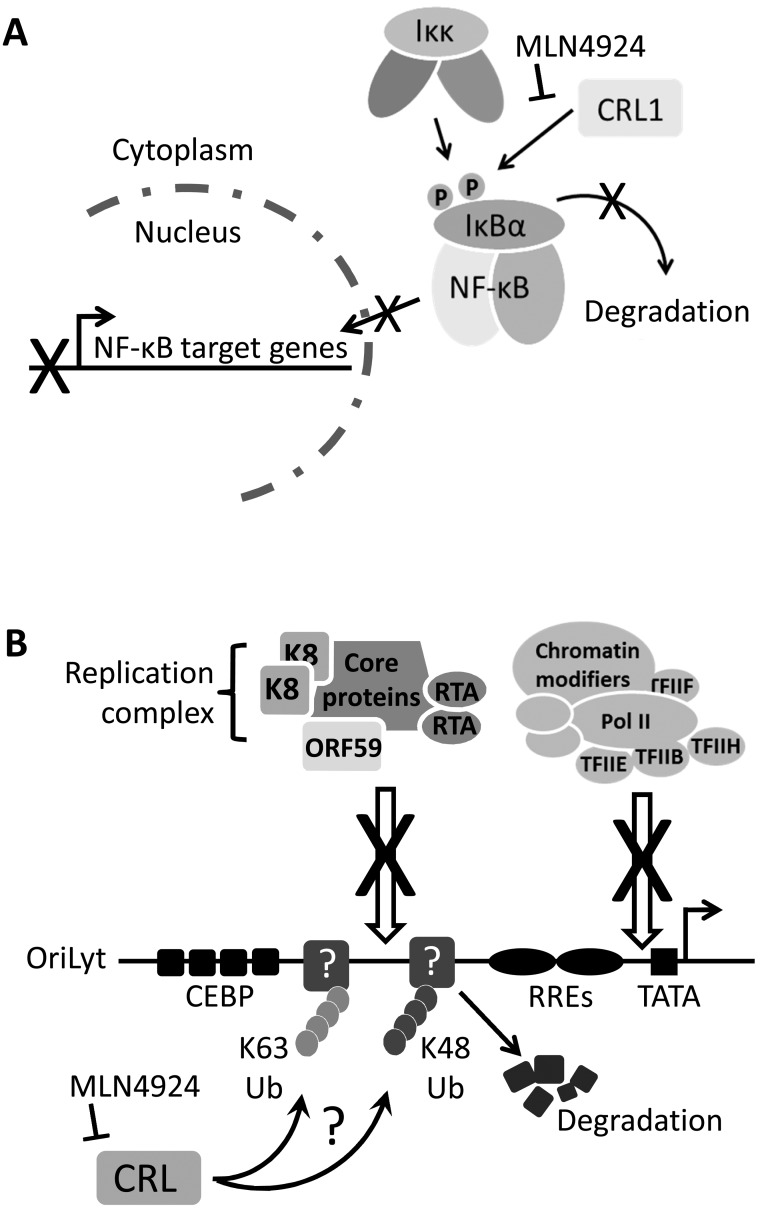
Summary. (**A**) NF-κB signaling is essential for the survival of PEL as it drives the expression of KSHV latency genes, suppresses lytic cycle-associated genes and promotes antiapoptotoic gene expression [28]. Normally, IκBα inhibits NF-κB by preventing its nuclear translocation. Upon stimulation, or modulation by viral proteins such as vFLIP, IκBα is phosphorylated which signals for the recruitment of the Cul1-containing βTrCP ubiquitin ligase, leading to its polyubiquitylation and degradation [32]. This releases NF-κB allowing it to translocate to the nucleus to activate transcription. This study has shown that MLN4924 blocks the degradation of IκBα thus preventing the nuclear translocation of NF-κB and subsequent PEL cell death. (**B**) The lytic cycle of KSHV is required for the pathogenesis of KS [7], and therefore the molecular mechanisms governing KSHV reactivation need to be understood. This study has shown that inhibiting NEDDylation prevents lytic reactivation by blocking the recruitment of the viral replication complex and the RNA polymerase II (Pol II) transcription complex at OriLyt. It is presently unknown how this is occurring, but it might suggest that unknown restriction factors at OriLyt are targeted by CRLs leading to their polyubiquitylation via K48-linked ubiquitin (thus targeting them for degradation) or K63-linked ubiquitin suggesting a CRL-mediated signaling requirement during lytic reactivation. Modification (either Ub or NEDD8) may also be required for the proper processing of proteins that are essential for reactivation. Nevertheless, this study has highlighted that NEDDylation may be a novel target for the treatment of various KSHV-associated malignancies.

The activation of NF-κB is central to KSHV infection (by modulating viral gene expression) and for the pathogenesis of KSHV-associated malignancies (via induction of inflammatory mediators and the expression of antiapoptotic genes). Inhibiting NEDDylation was clearly cytotoxic to PEL cells, and mechanistically, it appears that this was due to the inhibition of NF-κB. For example, we showed that MLN4924 led to: i) accumulation of pIκBα, ii) a KSHV gene expression profile consistent with inhibition of NF-κB and iii) the induction of apoptosis (Fig. 8). This corroborates recent work demonstrating that MLN4924 killed NF-κB-dependent ABC-DLBCL cells in a comparable fashion [27]. In fact, ABC-DLBCL cells were more sensitive than GCB-DLBCLs (that are not reliant on NF-κB) [27] suggesting that this drug may be more efficacious for cancers such as PEL and KS, where NF-κB activation is a requirement for malignancy. NF-κB is also required for transcription of the latency control locus which expresses an alternatively spliced transcript that produces *ORF71* (vFLIP), *ORF72* (vCyclin) and *ORF73* (LANA). As LANA is essential for maintenance of the KSHV genome during mitosis, NF-κB signaling is clearly required for sustained infection. Interestingly, MLN4924 did not appear to overtly affect LANA expression (despite reducing its mRNA level) which is in line with previous reports showing that its half-life exceeds the time course of our experiments [43] and signifying that cytotoxicity was not a result of KSHV genome loss. Moreover, the histological signature of KS involves elongated, spindle-shaped endothelial cells and vFLIP expression alone is sufficient to bring about this morphological change in endothelial cell cultures [44,45]. Importantly, this phenotypic alteration has been shown to be NF-κB-dependent; therefore, it will be of particular interest to investigate if MLN4924 can inhibit this characteristic feature of KS.

In contrast to its role in latency-associated transcription, NF-κB is a negative regulator of numerous lytic cycle-associated genes, including those analyzed in this study (*RTA*, *ORF57* and *ORF47*) [46]. The KSHV protein RTA is both necessary and sufficient for the induction of the lytic cycle, resulting in the expression of the entire KSHV genome and virion production. Many RTA-responsive genes contain RBP-Jκ binding sites, and the translocation of NF-κB into the nucleus allows it to bind RBP-Jκ and prevent its association with RTA. Hence, the induction of lytic cycle-associated gene expression in PEL and rKSHV.219 cells is very likely due to MLN4924-induced inhibition of NF-κB. The Cul1-containing βTrCP is the principal E3 ligase responsible for IκBα degradation [37]. In agreement the above hypothesis, we found that individual expression of dominant-negative versions of Cul1 (DNCul) was able to activate lytic cycle gene expression. Importantly, these data also suggest that MLN4924’s effect on viral gene expression was due to its inhibition of the CRL activity as opposed to a direct role of NEDDylation in KSHV gene expression or the over-arching effects a global block in NEDDylation would have on cellular function. However, an in-depth investigation of the various CRL components is required to fully elucidate the question of specificity.

In addition to the ability of DNCul1 to reactivate lytic cycle associated gene expression, DNCul4B and shRNA knockdown of Cul4B were both able to induce lytic cycle gene expression. Using *K12* expression as an indicator of latency versus lytic cycle-associated gene expression, the complete blockade of NEDDylation led to a reduction in its expression, whereas knockdown of Cul4B led to its increase, pointing to differing mechanisms. These data are in line with the hypothesis that complete blockade (via MLN4924) effects NF-κB expression by inhibiting CRL1 and that inhibition of Cul1 NEDDylation is more sensitive than its inhibition of Cul4B modification. It is not currently known whether differences exist in the sensitivity of individual CRLs following MLN4924 treatment. Of the known CRL functions, Cul4 activities are centered on chromatin regulation, such as chromosome condensation, heterochromatin formation and DNA replication and repair processes. Therefore, although Cul4’s potential role in the maintenance of KSHV latency is novel, it might not be surprising. The two Cul4 proteins are virtually identical apart from an extended N-terminal portion found in Cul4B that encodes a nuclear localization signal. Therefore, Cul4B predominantly resides in the nucleus, whereas Cul4A is recruited to the nucleus in response to genotoxic stress. This provides further credence to the hypothesis that Cul4B is important for the regulation of KSHV gene expression. The DNA damage response is a recurring theme in virus biology as it either aids or is a consequence of infection [24,47–49]. Cul4A and 4B play a pivotal role in the nucleotide excision repair (NER) process, and it was recently reported that human cytomegalovirus (HCMV) is dependent on NER during replication of its genome. Therefore, the role of Cul4 during KSHV infection merits further investigation.

A striking observation however, was that MLN4924 treatment blocked viral genome replication despite appreciable levels of viral gene expression. At 1 μM MLN4924, ORF57 expression was not significantly reduced, but genome replication was reduced by ca. 80%. We considered the possibility that the induction of apoptosis may be responsible for this, and so we repeated the experiments in the presence of a pan-caspase inhibitor (z-VAD-FMK). Even though this led to a recovery in KSHV protein expression even when up to 10 μM MLN4924 was used, genome replication was still not observed. Over the course of our studies, we did notice aberrant RTA localization in BCBL-1 cells treated with MLN4924, followed by reactivation (e.g. Fig. 7). Given that RTA’s function is to recruit the viral pre-replication complex, along with various cellular factors required for KSHV genome replication, its localization is intimately linked with sites of viral genome replication occurs. These sites are termed replication compartments (or replication centers) and can be observed as discrete foci at the nuclear periphery that co-stain with RTA and newly replicated viral DNA (this can be observed using BrdU or EdU that specifically marks viral DNA due to KSHV’s ability to block cellular DNA replication during reactivation). We therefore hypothesized that NEDDylation, or CRL-mediated ubiquitylation was required for KSHV genome replication (but not viral gene expression).

Firstly, we showed that reactivation of KSHV is associated with ubiquitylation within replication compartments, and that MLN4924 treatment inhibited this. We next investigated whether NEDDylation was required for RTA’s recruitment to the origins of lytic replication (OriLyt). Using ChIP analysis, we observed that treatment of latent cells led to an increase in Pol II (ca. 2-fold) and RTA (ca. 1.6-fold) occupancy, which agrees with our data showing that treatment led to activation of viral gene expression. Treatment followed by reactivation with Dox. however showed us that NEDDylation was required for RTA recruitment to OriLyt. Here we showed that 1 μM MLN4924 (a concentration that still permits viral gene expression) blocked RTA recruitment to OriLyt. Likewise, Pol II occupancy was reduced to similar levels. This confirmed to us that NEDDylation was required for the proper recruitment of the RTA (and therefore the pre-replication complex) to OriLyt, and offers and explanation for the aberrant localization of RTA and the inhibition of genome replication.

There are various hypotheses that may explain the importance of NEDDylation for the recruitment of the pre-replication complex to OriLyt (Fig. 8). It might be possible that proteins within the pre-replication complex are themselves targets for NEDD8 modification. We have addressed this for RTA and showed that RTA is not NEDDylated (S4 Fig); given that RTA is the protein responsible for recruitment, this result suggested that this hypothesis is unlikely [40]. It might also be possible that NEDDylation or CRL-mediated ubiquitylation of OriLyt-associated chromatin might be important for the recruitment of the pre-replication complex, as has recently been reported for the recruitment of repair enzymes during a DNA damage response [50]. Although it is still possible that these modifications play a role in cells, RTA binds to DNA in a sequence-specific manner, and it can do this *in vitro* in the absence of chromatin suggesting that this may not be the role of NEDDylation during reactivation [51]. A further hypothesis is that modification of a factor that resides at OriLyt during latency (in order to maintain latency) may be required during reactivation. This may include CRL-mediated ubiquitylation (via K48) that targets a latency-associated protein for degradation thus allowing access to OriLyt by the pre-replication complex. It may also involve Ub-modification via K63 linkage that might enhance binding *in vivo*. Various cellular proteins have been reported to interact with OriLyt during reactivation, but in that study, proteins that bound during latency were not investigated [42]. All of the above hypotheses merit further investigation but are unfortunately beyond the scope of this study.

In summary, we have demonstrated that NEDDylation is essential for various aspects of KSHV infection. Inhibition of NEDDylation using MLN4924 proved cytotoxic to PEL cells due to their dependence on NF-κB. Remarkably, we also show that a functioning NEDDylation cascade is essential for KSHV genome replication as it was required for the recruitment of the RTA-mediated pre-replication complex to OriLyt. These new findings have opened up new avenues of investigation regarding the regulation of herpesvirus latency and reactivation. Moreover, they demonstrate that inhibition of NEDDylation represents a novel approach for the treatment of KSHV-associated malignancies, including KS that is dependent on both lytic replication and the latency-associated activation of NF-κB.

## Materials and Methods

### Cell lines and drug treatments

BCBL-1 and BC-3 are primary effusion lymphoma (PEL) B cell lines latently infected with KSHV. TREx-BCBL-1-RTA cells (a kind gift of Dr. Jae Jung, University of Southern California) are a BCBL-1-based cell line that has been engineered to inducibly express exogenous Myc-tagged RTA by the addition of doxycycline, leading to a robust reactivation of the full KSHV lytic cycle [52]. The rKSHV.219 cell line maintains KSHV as a latent infection and was generated by infecting HEK293T cells with a recombinant KSHV that contains a constitutively active puromycin resistance and GFP gene, and an RFP gene that is fused to an RTA-responsive lytic cycle (*PAN*) promoter; hence, expression of RFP can be used as a reporter of RTA activity [53]. HEK293T cells were also used for co-immunoprecipitation experiments. All cells were maintained at 37°C in a humidified incubator with 5% CO_2_. The PEL cell lines were maintained in RPMI-1640 (Lonza) supplemented with 10% FBS (Life Technologies). TREx-BCBL-1-RTA media also contained 200 μg/ml hygromycin B (Life Technologies). The rKSHV.219 and HEK293T cell lines were maintained in DMEM (Lonza) supplemented with 10% FBS (Life Technologies). The rKSHV.219 cultures also contained 1 μg/ml puromycin. KSHV reactivation was induced in BCBL-1 and rKSHV.219 by the addition of 20 ng/ml 12-O-tetradecanoylphorbol 13-acetate (TPA) and 1.5 mM sodium butyrate (NaB) or 1 μg/ml doxycycline hyclate (Sigma) in TREx-BCBL-1-RTA cells.

MLN4924 [12] (Millennium Pharmaceuticals) stock solutions (10 mM) were prepared in DMSO and diluted in media prior to its addition to cells at the indicated concentrations, and for the indicated times. Routinely, 10^6^ cells in 12-well plates were treated. Inhibition of caspase enzymes was achieved by the addition of 50 μM z-VAD-FMK or FMK-negative control (MBL International) 30 min prior to MLN4924 and/or reactivation treatment. Inhibitors remained on the cells for the duration of the experiments.

### Viability assays

Cell viability was determined using the standard trypan blue exclusion method and using the ATPlite Luminescence ATP Detection Assay (Perkin Elmer). For the ATPlite assay, 10,000 cells were seeded into white 96-well plates and allowed to settle for 16 h at 37°C. Varying concentrations of MLN4924 were added to the cells followed by 96 h incubation at 37°C. Cellular proliferation was determined according to the manufacturer’s instructions.

### Cell cycle analysis

Cells (10^6^) were treated with 1 μM MLN4924 (or left untreated) for 24 h, washed in cold PBS and fixed for 24 h in cold 70% ethanol at -20°C. Prior to analysis, cells were washed in PBS and treated with 1 ml PBS, 10 μg/ml propidium iodide (Sigma), 0.5 mg RNase A for 3 h at 37°C. Cells were then pelleted, resuspended in 1 ml PBS and analysed using a Becton Dickinson BD-LSRFortessa flow cytometer. Data were fitted using ModFit software.

### Expression vectors and transfection

Most expression vectors were obtained from Addgene: pcDNA3-DN-hCul1-FLAG (plasmid 15818), pcDNA3-DN-hCul2-FLAG (plasmid 15819), pcDNA3-DN-hCul3-FLAG (plasmid 15820), pcDNA3-DN-hCul4A-FLAG (plasmid 15821), pcDNA3-DN-hCul4B-FLAG (plasmid 15822), pcDNA3-DN-hCul5-FLAG (plasmid 15823) [35], pcDNA3-HA-Cullin4A (plasmid 19907), pcDNA3-Myc-NEDD8 (plasmid 19943). pFLAG-CMV-4-NEDD8 was a kind gift from Dr Eric Stebbins (Rockefeller University) [54]. RTA expression vectors (pRTA and pRTA^H145L^) were gifts of Dr Gary Hayward (Johns Hopkins University). Cells were plated into 6-well plates and transfections routinely used 1 μg plasmid DNA and Lipofectamine 2000 (Life Technologies) following the manufacturer’s instructions.

### Knockdown of gene expression

Knockdown of Cul4B expression was accomplished using four individual SureSilencing shRNA expression vectors (Qiagen). TREx-BCBL-1-RTA cells (3 x 10^6^ per transfection) were transfected with 20 μg scramble shRNA (control) or 5 μg of each of four shCul4B vectors by nucleofection (Lonza; solution V and nucleofector program T-01). Four days post-transfection, RNA was extracted using Trizol (Life Technologies) and used to generate cDNA for qRT-PCR analysis (see below).

### Immunoblot analysis

Cells were washed in PBS and proteins extracted in lysis buffer containing 50 mM Tris (pH 7.4), 150 mM NaCl, 1% NP-40 and 1x protease inhibitor cocktail (Roche) for 15 min on ice and clarified by centrifugation at 12,000 x*g* for 10 min, 4°C. Cells used in Nuclear/cytoplasmic enrichments assays (ca. 10^6^) were washed in PBS, lysed in 50 μl PBS, 1% Triton X-100 and 1x protease inhibitor cocktail (Roche) for 15 min on ice and nuclei were pelleted at 2000 x*g* for 5 min. Cytoplasmic fractions (supernatant) were moved to new tubes and the nuclear pellets were washed three times in lysis buffer. SDS-PAGE and immunoblotting of normalized protein concentrations followed standard techniques using the following antibodies [55]: RTA, rabbit antisera (1:400), mouse mAb anti-Myc-tag (1:5000; Sigma), rabbit pAb anti-FLAG-tag (1:1000; Sigma), mouse mAb anti-HA-tag (1:5000; Life Technologies), mouse mAb anti-ORF57 (1:1000; Santa Cruz), rabbit mAb anti-Cullin 2 (1:1000; Life Technology), mouse mAb anti-PARP-1 (1:1000; Cell Signaling Technology), mouse mAb anti-GAPDH (1:5000; Sigma), mouse mAb anti-Lamin B (1:1000; Santa Cruz), rabbit mAb anti-Phospho-IκBα (ser32) (1:1000; Cell Signaling Technology), mouse mAb anti-p65/RelA (1:1000; Santa Cruz), rabbit mAb anti-Caspase 3 (8G10) (1:1000; Cell Signaling Technology), rabbit mAb anti-active Caspase 3 (Abcam; 1:250), mouse mAb anti-Caspase 9 (Cell Signaling Technology), mouse mAb anti-Cdt-1 (1:1000; Santa Cruz), mouse mAb anti-γH2Ax (ser139) (1:1000, Santa Cruz) rabbit mAb anti-Phospho-p53 (ser15) (1:1000; Cell Signaling Technology). Signals were detected using chemiluminescence and densitometry (ImageJ) was used to semi-quantify expression levels.

### Quantitative RT-PCR (qRT-PCR)

As previously reported [56], total cellular RNA was extracted from cells using Trizol (Life Technologies) according to the manufacturer’s instructions and contaminating DNA was removed using the DNA-*free* kit (Ambion). Complimentary DNA (cDNA) was generated from 1 μg RNA in 20 μl reaction volumes using M-MuLV reverse transcriptase (RT; NEB) according to the manufacturer’s recommendations with 5 ng oligo(dT). In parallel, negative control reactions were performed for each RNA by omitting RT in order to confirm that quantification represented cDNA and not contaminating DNA. Quantitative PCR reaction mixes (20 μl) included 1x SensiMix SYBR green master mix (Bioline), 0.5 μM each primer and 1 μl cDNA reaction mix. Cycling was performed in a RotorGene Q machine (Eppendorf) and included an initial 10 min denaturation step at 94°C, followed by 40 cycles of 30 s at 94°C, 30 s at 60°C and 30 s at 72°C. Melting curve analysis was performed between 65 and 95°C (with 0.2°C increments) to verify amplicon specificity. Quantification of *GAPDH* mRNA was used to normalize between samples, and the average cycle threshold (C_*T*_) was determined from three independent RNA samples from independent cultures. Relative expression compared to non-treated or DMSO-treated control cells was calculated using the ΔΔC_*T*_ method.

### Indirect immunofluorescence microscopy

Cells were cultured on poly-L-lysine coated coverslips in 12-well plates for 24 h at 37°C, gently washed with PBS, fixed using 4% formaldehyde (in PBS) for 10 min, permeabilized with PBS, 1% Triton X-100 for 10 min and washed three times with PBS as previously reported [57,58]. Where stated, some experiments involved the removal of soluble nuclear proteins (i.e. those not tightly bound to chromatin) using an “extraction first” method: here coverslips were treated (as previously reported [24]) with CSK buffer (10 mM PIPES, 100 mM NaCl, 300 mM sucrose, 3 mM MgCl_2_) containing 0.5% Triton X-100 for 2 min followed by 2 washes with CSK buffer alone. These cells were then fixed and processed as normal. Primary antibodies were diluted in PBS, 2% BSA, added to cells and incubated in humidity chambers for 2 h at 37°C or overnight at 4°C followed by 5 washed with PBS. The appropriate secondary antibodies (Alexa Fluor 488 or 594; Life Technologies) were diluted 1:500 in PBS, 2% BSA and incubated with cells for 1 h at 37°C followed by 5 washed with PBS. Coverslips were mounted in VECTORSHEILD with DAPI (Vectorlabs). Where experimentally possible, double staining was performed by incubating the coverslips with both primary antibodies, and following washing, both secondary antibodies at the same time. Images were captured using an LSM700 laser scanning microscope (Carl Zeiss) and processed using ZEN imaging software (Carl Zeiss). Antibodies included: RTA rabbit antisera (1:100), NF-κB RelA mouse mAb (1:250; Cell Signaling), anti-ubiquitinylated proteins clone FK2 mouse mAb (1:250; Millipore), mouse mAb anti RNA Pol II (Millipore).

### Virus reactivation assays

Virus reactivation was determined by two complimentary assays—identification of viral replication compartments by imaging the incorporation of EdU into replicating viral DNA and by quantitative PCR analysis of viral genome amplification. Incorporation of EdU was performed using a Click-iT EdU Imaging Kit (Life Technologies). Briefly, 1 x 10^6^ TREx-BCBL-1-RTA cells were drug-treated as appropriate, virus reactivation was induced by the addition of doxycycline and the cells were plated onto poly-L-lysine-coated coverslips. After 16 h, cells were pulsed for 45 min with 10 μM EdU, washed, fixed and permeablized according to the manufacturer’s recommendations. After detection of EdU (according to the manufacturer’s protocol), the cells were washed and further incubated with RTA antisera (1:100 in PBS, 2% BSA) for 1 h at 37°C, washed again and incubated with Alexa Fluor 488 goat anti-rabbit antibody (Life Technologies; 1:500 in PBS, 2% BSA) for 1 h at 37°C. Finally, DNA was stained using Hoechst 33342 (1:2000 in PBS) and mounted in VECTORSHIELD (Vectorlabs). Images were captured using an LSM700 laser scanning microscope (Carl Zeiss) using 639 nm, 488 nm and 405 nm lasers for the detection of EdU, RTA and DNA respectively and processed using ZEN imaging software (Carl Zeiss).

For quantitative PCR analysis 1 x 10^6^ TREx-BCBL-1-RTA cells were drug-treated as appropriate and virus reactivation was induced by the addition of doxycycline. At 24 h post-reactivation, total DNA was extracted using a DNA Minikit (Qiagen) and quantified by UV spectrophotometry. Viral DNA was quantified using 3.4 ng DNA, in 20 μl reaction volumes as described above for qRT-PCR, using primers specific for the ORF57 gene. Quantification of GAPDH was used to normalize between samples, and the average cycle threshold (C_*T*_) was determined from three independent samples from independent cultures. Relative levels of viral DNA was calculated using the ΔΔC_*T*_ method.

### Immunoprecipitation (NEDDylation assay)

HEK293T cells were plated into 6-well dishes and co-transfected with the indicated plasmids with pcDNA-Myc-NEDD8 or pFLAG-CMV-4-NEDD8 [54] for 24 h. Cells were washed in PBS and proteins extracted in 1 ml lysis buffer (see above) and incubated with anti-c-Myc agarose (Sigma) or goat anti-DDDDK conjugated agarose (for detection of FLAG-tagged proteins; Abcam) following the manufacturer’s recommendations. Immunoprecipitated (NEDDylated) proteins were eluted in Laemmli buffer and subject to immunoblot analysis (see above).

### Chromatin immunoprecipitation (ChIP)

Chromatin immunoprecipitation (ChIP) assays were carried out as previously stated [59] using the EZ-ChIP chromatin immunoprecipitation kit (Millipore). Per condition, 10^7^ cells were treated with 1% formaldehyde for 10 min prior to three washes with ice-cold PBS. Cells were then resuspended in 3 ml SDS lysis buffer (1% SDS, 10 mM EDTA, 50 mM Tris, [pH 8.1]) containing 1x protease inhibitor cocktail II and sonicated 15 time with 20 s pulses at 4°C. Insoluble material was removed by centrifugation for 10 min at 12,000 x*g*, 4°C. For each immunoprecipitation (IP), 100 μl of prepared chromatin was added to 900 μl of dilution buffer (0.01% SDS, 1.1% Triton X-100, 1.2 mM EDTA, 16.7 mM Tris-HCl, [pH 8.1], 167 mM NaCl) containing 1x protease inhibitor cocktail II. The lysates were then pre-cleared with Protein G agarose before being incubated with appropriate antibodies (Myc-tag and RNA Pol II) overnight at 4°C. The following day 60 μl of Protein G agarose was added to each IP and rotated for 1 h at 4°C. The Protein G agarose-antibody/chromatin complexes were then washed sequentially in 1 ml of the provided buffers; low salt immune complex wash buffer (0.1% SDS, 1% Triton X-100, 2 mM EDTA, 20 mM Tris-HCl, [pH 8.1], 150 mM NaCl), high salt immune complex wash buffer (0.1% SDS, 1% Triton X-100, 2 mM EDTA, 20 mM Tris-HCl, [pH 8.1], 500 mM NaCl), LiCl immune complex wash buffer (0.25 M LiCl, 1% IGEPAL CA630, 1% deoxycholic acid sodium salt, 1 mM EDTA, 10 mM Tris, [pH 8.1]) and TE buffer (10 mM Tris-HCl, [pH 8.0], 1 mM EDTA). The DNA/protein complexes were eluted in 200 μl elution buffer (1% SDS, 0.1 M NaHCO_3_) before reversal of crosslinks with 5 M NaCl for 4 h at 65°C. Samples were then treated with RNase A at 37°C for 30 min followed by incubation with Proteinase K for 2 h at 45°C. The resulting DNA was purified over the provided spin filter columns, with elution in 50 μl of elution buffer C. The purified DNA was then subject to qPCR amplification using primers directed to the OriLyt (Forward primer: 5’- ACG GGC CTG GAA TCT CGC CTC TGG-3’ and Reverse primer: 5’- ATG GGC GTA ACC GTA GGA CAA GCT G-3’). Each qPCR reaction performed in triplicate containing 5 μl purified DNA and was quantified as described above.

### Oligonucleotides

Oligonucleotide primer sequences are available upon request.

## Supporting Information

S1 Fig Cullin proteins are NEDDylated in KSHV-infected cells. NEDDylation assays were carried out in latently-infected rKSHV.219 cells. Cells were transfected with FLAG-NEDD8 along with the indicated Myc-tagged protein. FLAG-NEDDylated proteins were immunoprecipitated and modified proteins were detected by immunoblot analysis using αMyc antibodies. Myc-Hey1, a protein not expected to be NEDDylated was used as a negative control.
(TIF)

S2 Fig MLN4924-induced inhibition of NF-κB. Immunofluorescence (confocal) analysis NF-κB in untreated TREx-BCBL-1-RTA cells maintaining a latent infection (top left) showed low levels of NF-κB subunit RelA (p65) in the nucleus—a requirement for latency-associated gene expression and PEL viability. Upon lytic reactivation (dox-treated), RelA translocated to the nucleus (top right) due to the lytic cycle-associated downregulation of IκBα [34]. In 10 μM MLN4924-treated latent cells (bottom left), the nucleus was devoid of RelA in most cells supporting the hypothesis that MLN4924 stabilized IκBα, thus preventing the nuclear translocation of NF-κB (see Fig. 3); however, in MLN4924-treated cells (10 μM) where the lytic cycle was induced (bottom right), RelA still readily translocated to the nucleus, further highlighting the role of IκBα stabilization for the MLN4924-associated inhibition of NF-κB signaling.
(TIF)

S3 Fig MLN4924 prevents the proper organization of replication compartments. Upon dox-induced reactivation of the KSHV lytic cycle in TREx-BCBL-1-RTA cells, the proteins required for KSHV gene expression and genome replication are recruited to discrete foci known as replication compartments (arrows, top panels). These include various viral proteins (such as RTA—red) and cellular factors (e.g. RNA Pol II—green). Treatment of cells with 1 μM MLN4924 (here for 16 h) prevents the proper organisation of KSHV replication compartments (bottom panels). NT denotes no treatment.
(TIF)

S4 Fig RTA is not NEDDylated. NEDDylation assays were carried out in transfected HEK293T cells. Target proteins were expressed in the presence of Myc-NEDD8, and anti-Myc agarose was used to immunoprecipitate NEDDylated proteins. HA-Cul4A and HA-PP2A served as positive and negative controls, respectively. Expression of RTA or the RTA^H145L^ mutant was detected using RTA antisera.
(TIF)
