# Supplementary figures and images for: NEDDylation Is Essential for Kaposi’s Sarcoma-Associated Herpesvirus Latency and Lytic Reactivation and Represents a Novel Anti-KSHV Target
Source: PLoS Pathog. 2015 Mar 20;11(3):e1004771. doi: 10.1371/journal.ppat.1004771 (PMC4368050; doi:10.1371/journal.ppat.1004771)

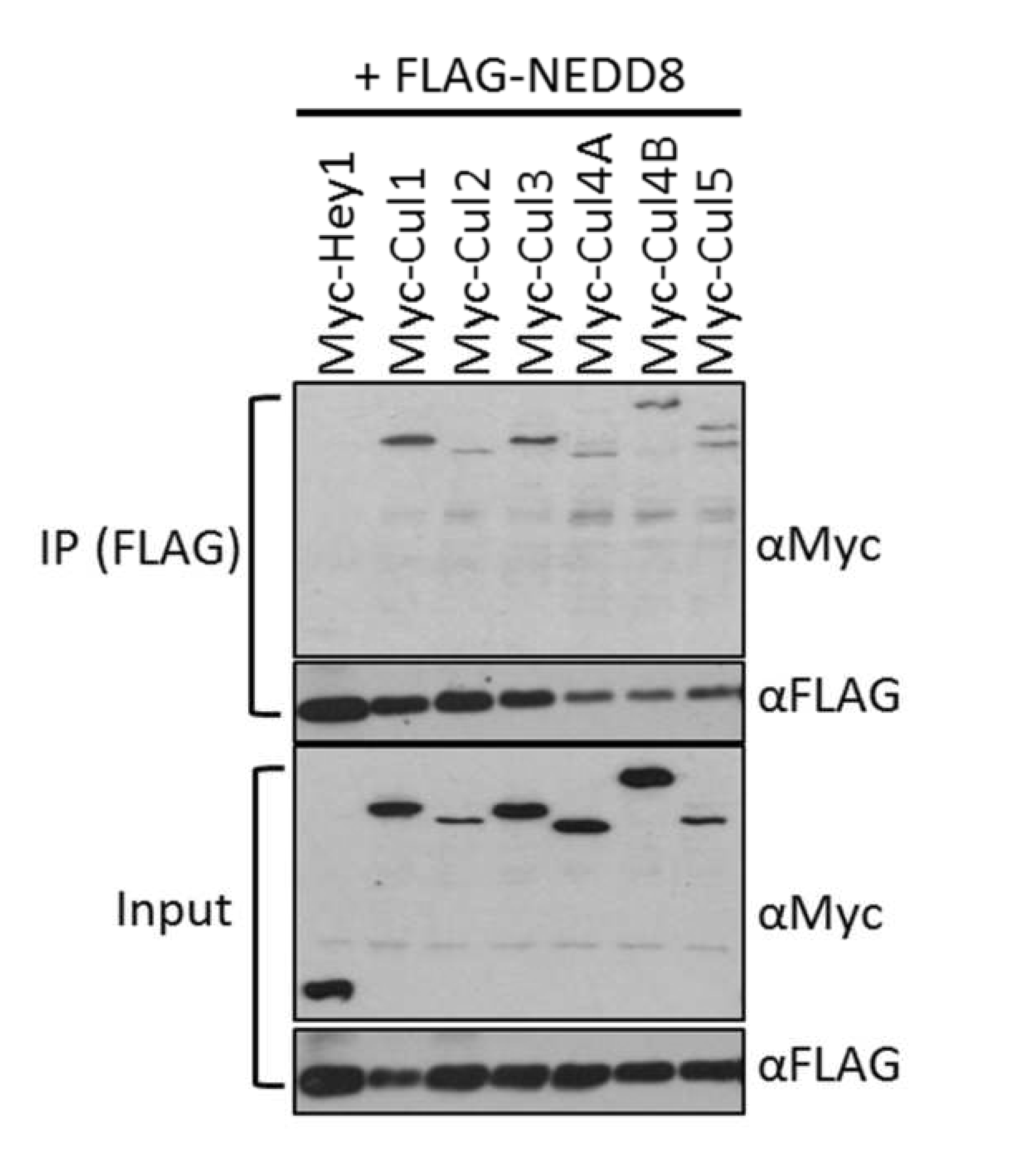

Supplement: S1 Fig — NEDDylation assays were carried out in latently-infected rKSHV.219 cells. Cells were transfected with FLAG-NEDD8 along with the indicated Myc-tagged protein. FLAG-NEDDylated proteins were immunoprecipitated and modified proteins were detected by immunoblot analysis using αMyc antibodies. Myc-Hey1, a protein not expected to be NEDDylated was used as a negative control. (TIF) [file ppat.1004771.s001.tif]

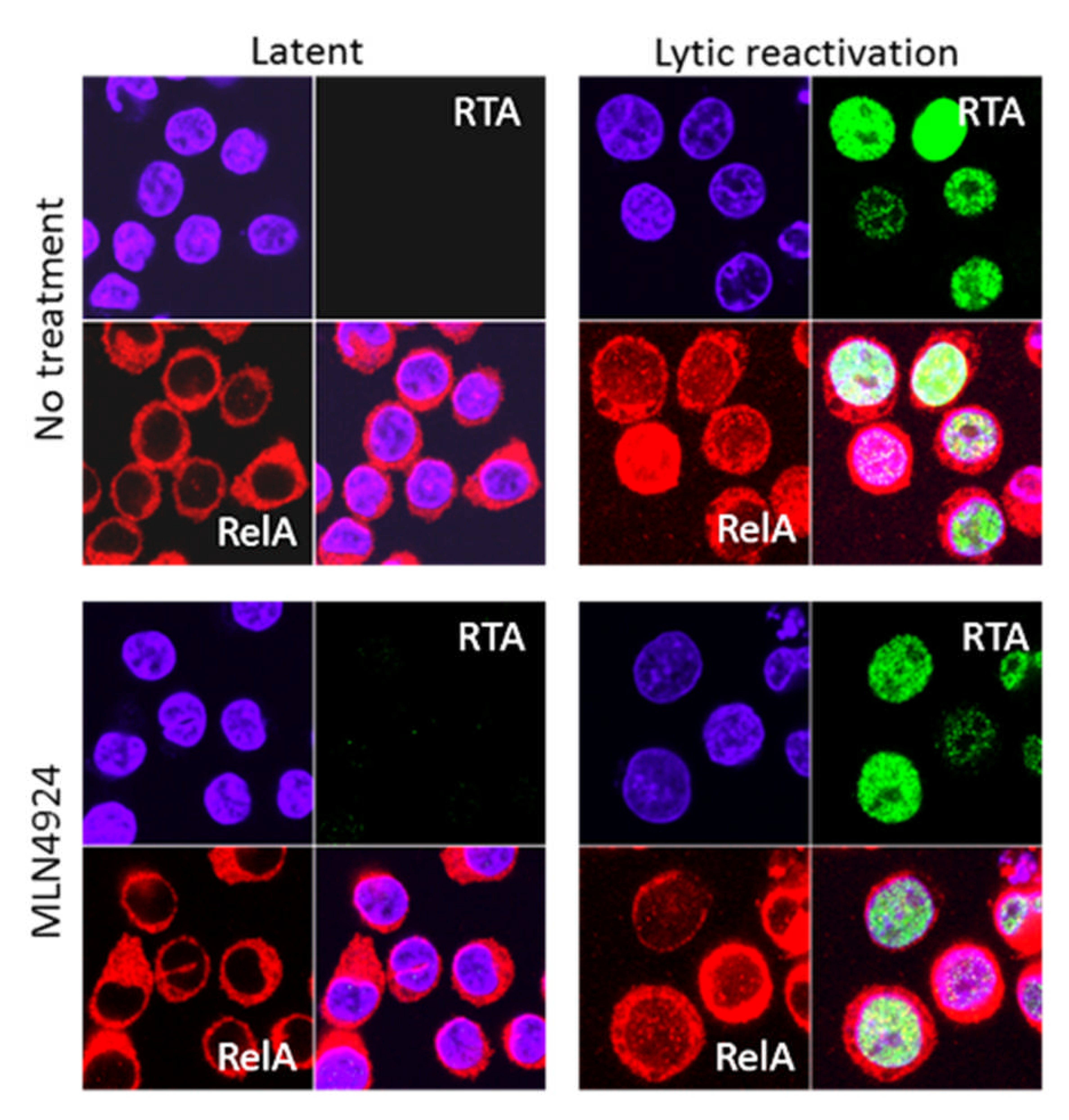

Supplement: S2 Fig — Immunofluorescence (confocal) analysis NF-κB in untreated TREx-BCBL-1-RTA cells maintaining a latent infection (top left) showed low levels of NF-κB subunit RelA (p65) in the nucleus—a requirement for latency-associated gene expression and PEL viability. Upon lytic reactivation (dox-treated), RelA translocated to the nucleus (top right) due to the lytic cycle-associated downregulation of IκBα [34]. In 10 μM MLN4924-treated latent cells (bottom left), the nucleus was devoid of RelA in most cells supporting the hypothesis that MLN4924 stabilized IκBα, thus preventing the nuclear translocation of NF-κB (see Fig. 3); however, in MLN4924-treated cells (10 μM) where the lytic cycle was induced (bottom right), RelA still readily translocated to the nucleus, further highlighting the role of IκBα stabilization for the MLN4924-associated inhibition of NF-κB signaling. (TIF) [file ppat.1004771.s002.tif]

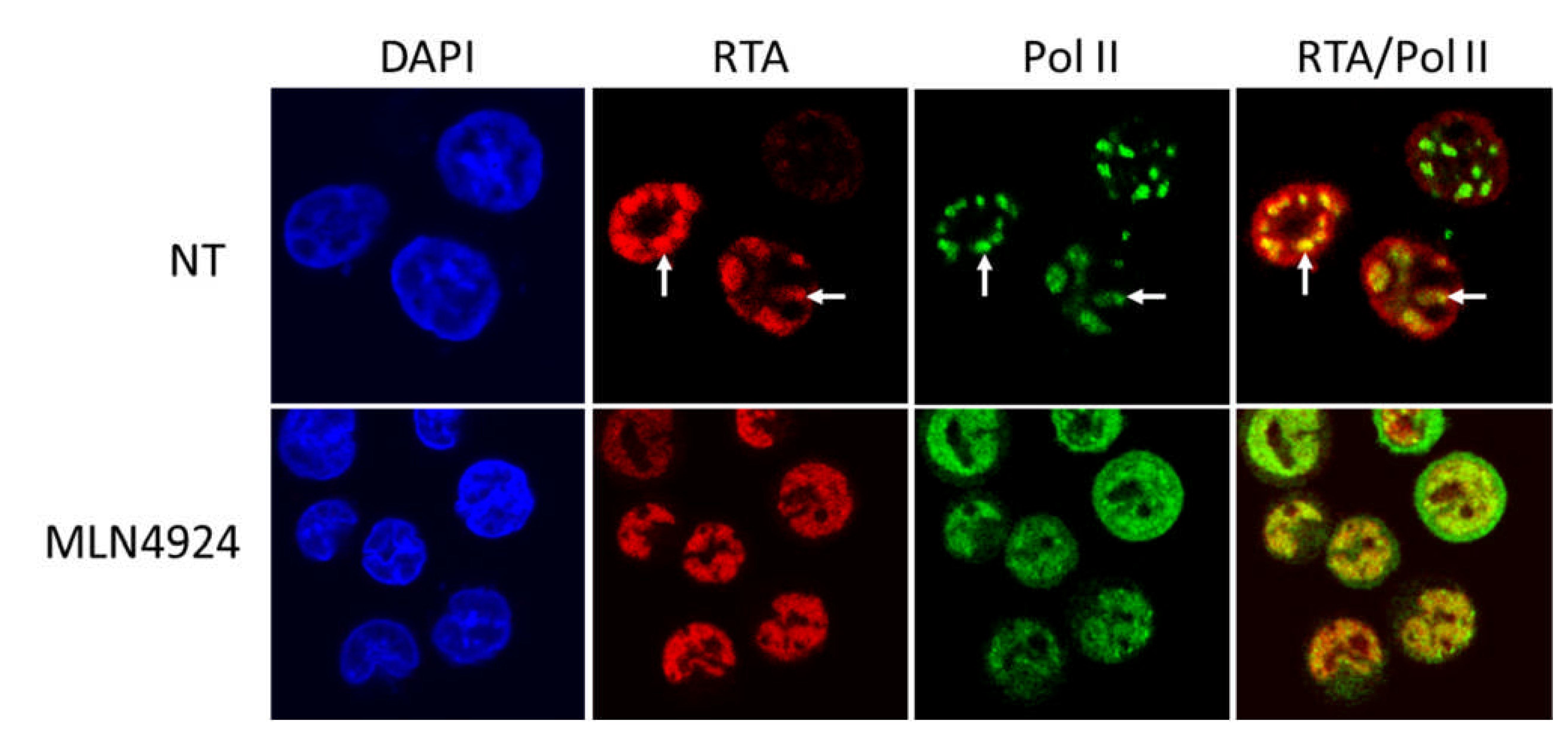

Supplement: S3 Fig — Upon dox-induced reactivation of the KSHV lytic cycle in TREx-BCBL-1-RTA cells, the proteins required for KSHV gene expression and genome replication are recruited to discrete foci known as replication compartments (arrows, top panels). These include various viral proteins (such as RTA—red) and cellular factors (e.g. RNA Pol II—green). Treatment of cells with 1 μM MLN4924 (here for 16 h) prevents the proper organisation of KSHV replication compartments (bottom panels). NT denotes no treatment. (TIF) [file ppat.1004771.s003.tif]

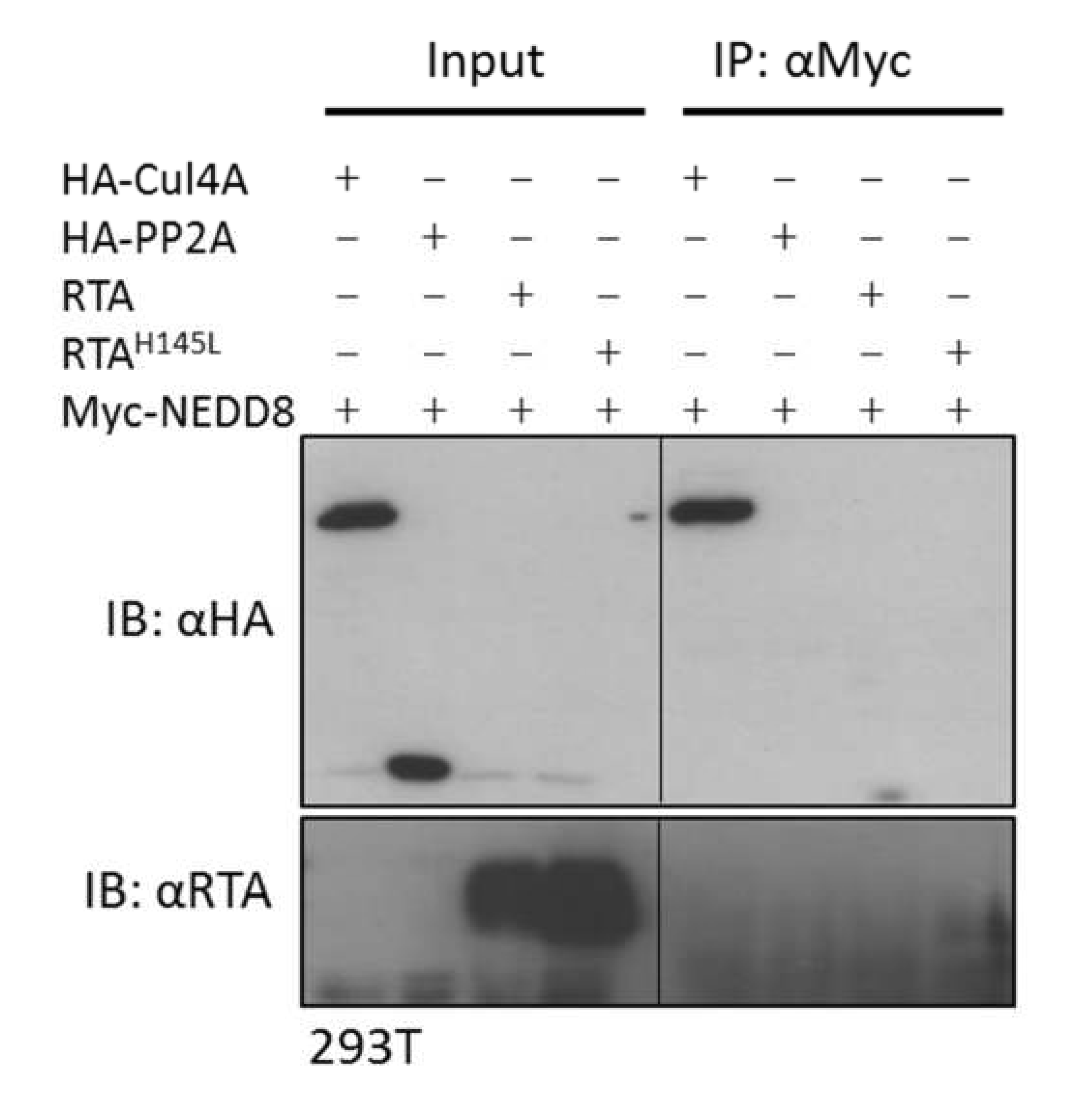

Supplement: S4 Fig — NEDDylation assays were carried out in transfected HEK293T cells. Target proteins were expressed in the presence of Myc-NEDD8, and anti-Myc agarose was used to immunoprecipitate NEDDylated proteins. HA-Cul4A and HA-PP2A served as positive and negative controls, respectively. Expression of RTA or the RTAH145L mutant was detected using RTA antisera. (TIF) [file ppat.1004771.s004.tif]
